# Persistence of *Listeria monocytogenes* ST5 in Ready-to-Eat Food Processing Environment

**DOI:** 10.3390/foods11172561

**Published:** 2022-08-24

**Authors:** Xin Liu, Wenjie Chen, Zhixin Fang, Ying Yu, Jing Bi, Jing Wang, Qingli Dong, Hongzhi Zhang

**Affiliations:** 1 School of Health Science and Engineering, University of Shanghai for Science and Technology, Shanghai 200093, China; 2 Shanghai Municipal Center for Disease Control and Prevention, Shanghai 200336, China; 3 The Minhang District Center for Disease Control and Prevention, Shanghai 201100, China

**Keywords:** *Listeria monocytogenes*, persistence, whole genome sequencing, biofilm formation, chlorine-containing disinfectant

## Abstract

Most human listeriosis is foodborne, and ready-to-eat (RET) foods contaminated by *Listeria monocytogenes* during processing are found to be common vehicles. In this study, a total of four *L. monocytogens* STs (ST5, ST121, ST120, and ST2) have been identified in two RTE food plants from 2019 to 2020 in Shanghai, China. The *L. monocytogenes* ST5 was predominant in one RTE food processing plant, and it persists in the RTE meat processing plant with continued clone transmission. The genetic features of the four STs isolates were different. ST5 and ST121 had the three genes *clpL*, *mdrL*, and *lde*; however, ST120 and ST2 had two genes except for *clpL*. SSI-1was present in ST5, ST121, and ST120. Additionally, SSI-2 was present only in the ST121 isolates. ST120 had all six biofilm-forming associated genes (*actA*, *prfA*, *lmo0673*, *recO*, *lmo2504* and *luxS*). The ST2 isolate had only three biofilm-forming associated genes, which were *prfA*, *lmo0673*, and *recO*. The four ST isolates had different biofilm formation abilities at different stages. The biofilm formation ability of ST120 was significantly higher when grown for one day. However, the biofilm formation ability of ST120 reduced significantly after growing for four days. In contrast, the biofilm formation ability of ST5 and ST121 increased significantly. These results suggested that ST5 and ST121 had stronger ability to adapt to stressful environments. Biofilms formed by all four STs grown over four days can be sanitized entirely by a disinfectant concentration of 500 mg/L. Additionally, only ST5 and ST121 biofilm cells survived in sub-lethal concentrations of chlorine-containing disinfectant. These results suggested that ST5 and ST121 were more resistant to chlorine-containing disinfectants. These results indicated that the biofilm formation ability of *L. monocytogenes* isolates changed at different stages. Additionally, the persistence in food processing environments might be verified by the biofilm formation, stress resistance, etc. Alternatively, these results underlined that disinfectants should be used at lethal concentrations. More attention should be paid to ST5 and ST121, and stronger surveillance should be taken to prevent and control the clonal spread of *L. monocytogenes* isolates in food processing plants in Shanghai.

## 1. Introduction

*Listeria monocytogenes* is an important foodborne pathogen, which causes listeriosis [1]. Most human listeriosis is foodborne, and the most popular vectors are ready-to-eat (RTE) foods, such as meat, dairy products, seafood and fresh produce, which are susceptible to contamination by *L. monocytogenes* strains in factory processing environments [2]. Previous studies suggested that *L. monocytogenes* strains can be introduced into food processing facilities and food products through raw materials by cross-contamination [3]. Furthermore, several reports have indicated that *L. monocytogenes* can persist in food processing environments for a long time, even up to ten years [4,5]. Different STs *L. monocytogenes* have different adaptation abilities to environments [6,7,8]. It has been reported that ST121, ST9, ST8, ST7, ST155, ST177, and ST204 have a special adaptation to environments and food matrices [9,10,11,12]. Among the great diversity of *L. monocytogenes* strains from food production, ST5 strains have been reported to be contaminated food processing environments [13,14], which is consistent with our previous studies.

In previous study, we characterized *L. monocytogenes* strains isolated from two RTE meat processing plants in the Shanghai municipal area, China, during 2019–2020 by pulsed-field gel electrophoresis and whole genome sequencing [6]. A total of 29 *L. monocytogenes* have been isolated. Of the 29 isolates, 21 were from the plant A and eight of 22 isolates were from the plant B. The *L. monocytogenes* ST5 isolates with the same pulsotype were predominant (72.4%, 21/29), which were identified in processing environments, even in an end-product at plant A. In addition, core genome multilocus sequencing typing identified up to nine allelic differences, with the most closet pairwise differences among these ST5 isolates included 0–16 small nucleotide polymorphisms. Therefore, *L. monocytogenes* ST5 isolates were thought to persist in plant A and continue clonal transmission from 2019 to 2020. In contrast, no *L. monocytogenes* ST5 isolates were found in the processing environments of plant B. Other STs isolates (ST120 and ST2) were detected only once in 2019 in plant A and were defined as transient isolates. Three *L. monocytogenes* ST121 isolates were detected in raw materials from plant B, and there was one *L. monocytogenes* ST121 strain being isolated from processing facility of plant A in 2020. It has been reported that *L. monocytogenes* ST121 isolates have persisted in food processing environments for a long time [15]. Therefore, in this study, the four STs (ST5, ST2, ST120 and ST121) were used to compare and analyze the persistent mechanism of *L. monocytogenes* ST5 isolates in food processing environments.

Whole-genome sequence (WGS) analysis of *L. monocytogenes* strains has revealed a high degree of similarity in both gene content and tissue structure [16,17,18]. However, many studies have shown that the *L. monocytogenes* strains were diverse in terms of general virulence, ecology, and physiology [19,20]. These results suggested that a relatively small number of distinct regions might play an important role in the persistence of different *L. monocytogenes* strains, such as resistance markers on plasmids, prophages (e.g., pLM33), stress-associated genes (e.g., SSI-1, SSI-2), biofilm-forming associate genes (e.g., *luxS*), and tolerance to disinfectant associated genes (e.g., *actA*, *prfA*) [21,22]. Previous surveillance has revealed that the *L. monocytogenes* ST5 has persisted in an RTE food processing plant during 2019–2020 [6]. Thus, how to control the occurrence of *L. monocytogenes* in food processing environments has become a major task for food safety. Previously, many studies have focused on the mechanism of survival of *L. monocytogenes* strains in food processing environments. The prevalence and persistence of *L. monocytogenes* strains in food processing environments occur mainly due to biofilm formation, persistence, resistance to disinfectants and virulence, and reproducing at low temperatures [7,8]. Therefore, in this study, four STs (ST5, ST120, ST2 and ST121) of *L. monocytogenes* from the two RTE food processing plants in Shanghai have been analyzed. The aims of this study were (1) to identify the key genome features contributing to persistence in RTE meat processing environments for a long time in China; (2) to assess the ability of resistance in RTE food processing environments by biofilm forming and tolerance to disinfectants; and (3) to provide a basis for measurement to prevent and control the persistence of *L. monocytogenes* in RTE processing environments.

## 2. Materials and Methods

### 2.1. Genomic DNA Extraction and WGS

The four STs *L. monocytogenes* isolates from two RTE meat processing plants were used in this study, including 16 ST5 isolates from processing environments and end products, four ST121 isolates from raw material and processing environments, one ST2 isolate from accessory material, and one ST120 isolate from raw material.

Four *L. monocytogenes* isolates were cultured overnight. Genomic DNA was extracted using a DNeasy Blood & Tissue Kit (QIAGEN, Hilden, Germany) following the manufacturer’s protocol with minor changes. The cells were pre-lysed with lysozyme for 30 min at 37 °C, and the proteinase K treatment was extended to 30 min. DNA concentration, quality, and integrity were identified using a NanoDrop Spectrophotometer (Thermo Scientific, Waltham, MA, USA). Sequencing libraries were generated using the TruSeq DNA Sample Preparation Kit (Illumina, USA). Then, genome sequencing was conducted using the Illumina Hiseq platform (Illumina). Finally, the reads were trimmed and assembled using the CLC Genomics Workbench v7.0 (CLC Bio, Aarhus, Denmark), and the assembled overlap clusters were exported as raw sequencing reads.

These reads were checked using FastQC version (v) 0.11.2 (Cambridge, London, UK) and trimmed using Trimmomatic v 0.36. Then, the trimmed reads were assembled using BioNumerics v 7.6 (Applied Maths, Kortrijk, Belgium), and the assembled sequence was used for further analysis.

### 2.2. Serogroup and ST Determination

All five specific genes, including *lmo*0737, *lmo*1118, *ORF2819*, *ORF2110*, and *prs*, were used to determine the serogroup of *Listeria monocytogenes* [23]. The sequence data of the five genes were obtained based on WGS data. Furthermore, the serogroup was identified by BioNumerics software (version 7.6 Applied Maths, Kortrijk, Belgium).

STs were determined using BioNumerics software based on the classical seven housekeeping loci MLST schemes (*abcZ*-*bglA*-*cat*-*dapE*-*dat*-*ldh*-*lhkA*) [23], and the sequence data of the isolates were obtained from the genomic data.

### 2.3. Prediction of Stress-Related Genes, Biofilm-Forming Related Genes and Disinfectant Resistant Genes

Genome assemblies were screened for the presence/absence of stress-related genes and biofilm-forming associated genes using DNAstar (7.1). These genes referred to previous studies and were also retrieved from the NCBI [24,25]. All alleles for stress tolerance were recovered from the *Listeria* database hosted by the Pasteur Institute, Paris, France (https://bigsdb.pasteur.fr/listeria). (accessed on 10 September 2021).

### 2.4. Prediction of Prophages and Plasmids

To identify putative prophages, the PHASTER (www.phaster.ca) (accessed on 12 September 2021). server was used to search for genome assemblies [25]. The application evaluates prophage regions as “*intact*”, “*questionable*”, or “*incomplete*” based on the criteria, such as the number of coding sequences (CDSs) homologous to some phages and the percentages of CDSs matching a particular phage. Intact and problematic regions with sequence lengths longer than 20 kbp were used for the prophage analysis.

The plasmids were predicated using PlasmidFinder (https://cge.cbs.dtu.dk/services/PlasmidFinder/) (accessed on 12 September 2021) and a minimum nucleotide sequence identity of 90% [26].

### 2.5. Biofilm-Forming Test

The four STs *L. monocytogenes* were analyzed in a biofilm-forming test, which were LM19052 (ST5), LM2061 (ST121), LM1964 (ST120), and LM19053 (ST2). Each experiment included three technical replicates and was repeated twice using independent bacterial cultures.

The biofilm formation assay was performed according to the method of Zhang et al. [6]. Stainless steel coupons (304, 1.2 cm × 1.4 cm × 0.1 cm) were immersed in acetone solution for 3 h. After being wiped, these coupons were soaked overnight in 75% (*v*/*v*) ethanol and rinsed with distilled water. Finally, they were autoclaved at 121 °C for 15 min before using.

Biofilm was cultured on sterile coupons in tryptic soy broth with yeast extract (TSB-YE). First, the *L. monocytogenes* were inoculated into 10 mLTSB-YE and incubated overnight at 37 °C. Second, the overnight culture was gradient diluted with TSB-YE to a final concentration of 10^4^ CFU/mL, and then, 2 mL of the dilution was dispensed into a 24-well polystyrene plate (Thermo Scientific™, Waltham, MA, USA) with one clean stainless steel coupon placed in each well. Finally, the samples were then incubated at 25 °C for 1–5 days, and the coupons were removed at regular intervals each day. The cultured stainless steel coupons were washed with 3 mL of 0.85% sodium chloride to remove loose cells and subsequently vortexed for 1 min in a tube containing 10 mL of 0.85% sodium chloride and sterile glass beads to shake off the attached biofilm cells for counting.

### 2.6. Disinfectant Tolerance

The disinfection of biofilms was performed as previously described with minor modification [27]. After the preparation of a biofilm, either firmly attached or grown as biofilm, as described above, the coupons were removed using sterile tweezers and washed with 3 mL 0.85% sodium chloride to remove loose cells. Then, the coupon was separately placed in a 24-well polystyrene plate for treatment with 2 mL of 125, 250 and 500 mg/L chlorine-containing disinfectant for 30 s and 60 s, respectively at room temperature. A coupon was placed in a 2 mL 0.85% sodium chloride for untreated control. Next, the coupons were placed into glass tubes with 5 mL sodium thiosulfate/neutralization broth (replacing D/E neutralization broth) and glass beads for ten minutes. Finally, after vortexing for 1 min, the liquid was used to enumerate the viable biofilm cells in TSA-YE. Plate counts were converted to logCFU/cm^2^, and the logarithmic reductions (logCFU/cm^2^) of live cells after disinfection were calculated by subtracting the survivors’ log in 0.85% sodium chloride (negative control). Each experiment included three technical replicates and was repeated thrice using independent bacterial cultures.

### 2.7. Statistical Analysis

Experimental data on biofilm formation and disinfectants were analyzed by the analysis of variance (ANOVA) procedure using SPSS^TM^ (IBM^®^ Version 22, Armonk, NY, USA). Tukey’s post hoc test was used to determine the significant differences in mean values with significance considered at *p* < 0.05.

## 3. Results

### 3.1. STs Distribution of L. monocytogenes Strains from RTE Food Processing Plants

**Figure 1:**
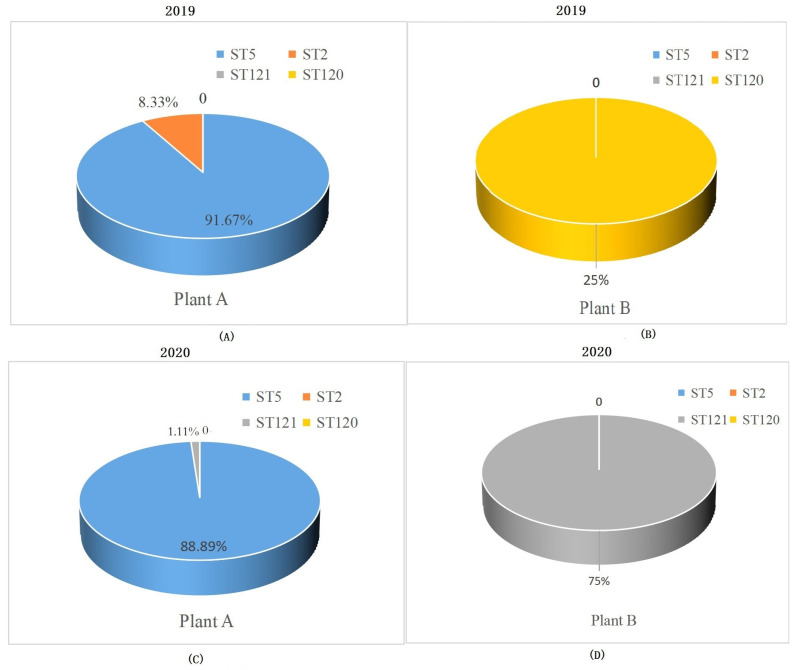
The distribution of the four STs in two RTE food processing plants in this study. (**A**): The distributions of four STs in plant A in 2019. (**B**): The distribution of four STs in plant B in 2019. (**C**): The distribution of four STs in plant A in 2020. (**D**): The distribution of four STs in plant B in 2020. Different colors represent different proportions of different ST.

The four ST isolates have been identified from the two RTE meat food processing during 2019–2020. The distribution of ST *L. monocytogenes* isolates in plant A and B was different. In plant A, the ST5 isolates were prevalent, accounting for 91.6% in 2019 and 88.89% in 2020 (Figure 1A,B). ST2 isolates (8.33%) were identified in 2019 and ST121 isolates (1.11%) were identified in 2020 in plant A. Only ST120 and ST121 isolates (one and three *L. monocytogenes*, respectively) have been identified in plant B (Figure 1C,D).

### 3.2. Genetic Features of Four STs L. monocytogenes in the Two RTE Meat Food Processing Plants

To investigate the genetic relatedness of *L. monocytogenes* with the ability of persistence in a food processing plant, the genetic features of four *L. monocytogenes* belonging to the four STs isolates were analyzed, such as serogroups, stress survival Islet, plasmids, *inlA* and prophages (Table 1). The four *L. monocytogenes* isolates were identified as three serogroups, which were ST5-IIb (1/2b, and 3b.7), ST121-IIa (serotypes 1/2a, 3a, and 3c), ST120-IIa (serotypes 1/2a, 3a, and 3c), and ST2-IVb (4b, 4d, and 4e), respectively. The genetic features of the same STs isolates were similar. SSI-1was present in ST5, ST121, and ST120. Additionally, SSI-2 was present only in the ST121 isolates. The three STs (ST5, ST121, and ST120) isolates had intact *inlA*. However, ST2 had a truncated *inlA*. Two plasmids were identified; they were pLM33 and pLM5578. The ST2 isolate had the two plasmids, ST5 isolates had pLM33, and ST121 isolates had pLM5578. However, ST120 isolates had no plasmids. Only one intact prophage was identified. The ST5 and ST2 isolates had the same prophage B025_NC_009812.

### 3.3. Biofilm-Forming Associated Genes and the Biofilm-Forming Ability of Four L. monocytogenes STs Isolates

The scanning of six biofilm-forming-associated genes (*actA*, *prfA*, *lmo0673*, *recO*, *lmo2504* and *luxS*) revealed that the presence in the four STs isolates was different. ST120 had all six biofilm-forming associated genes, which was followed by ST5 isolates, which had five genes except for *actA*. The S121 isolates had four biofilm-forming-associated genes except for *actA* and *prfA*. The ST2 isolate had only three biofilm-forming-associated genes, which were *prfA*, *lmo0673*, and *recO*. The ST2 had no *actA*, *lmo2504* and *luxS* genes (Table 1).

**Figure 2:**
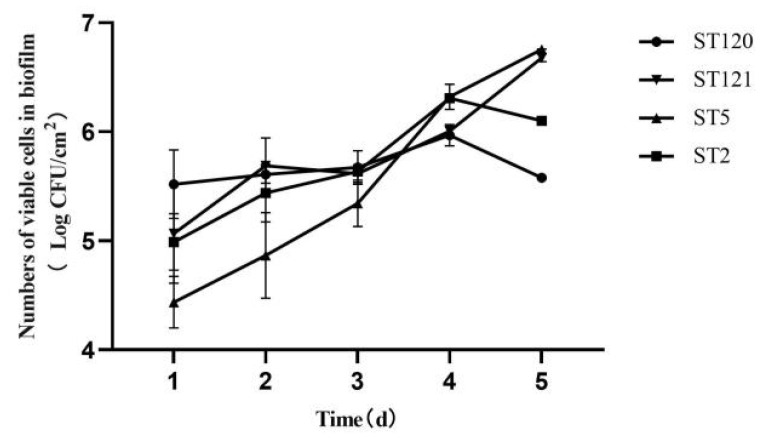
Trends of biofilm formation of four ST isolates during five days. Round represents ST120, upside down triangle represents ST121, triangle represents ST5, and square represents ST2.

As shown in Figure 2, the enumeration of biofilm cells of the four ST isolates was different from time to time (Figure 1). The biofilm cells of the STs isolates increased from the first day to the fifth day, and the enumeration of biofilm cells increased from 4.44 to 6.76 log. The biofilm cells of the ST121 isolates increased from 5.07 to 6.68 log during the five days, except that biofilm cells minor reduced from 5.69 to 5.62 log from the second day to the third day. The biofilm cells of the ST120 isolate increased from 5.52 to 5.97 log during four days and then reduced from 5.97 to 5.58 log in the fifth day. The same trend was found in the ST2 isolate. The biofilm cells of the ST2 isolate increased from 4.99 to 6.31 log during four days and then reduced from 6.31 to 6.10 log on the fifth day.

**Figure 3:**
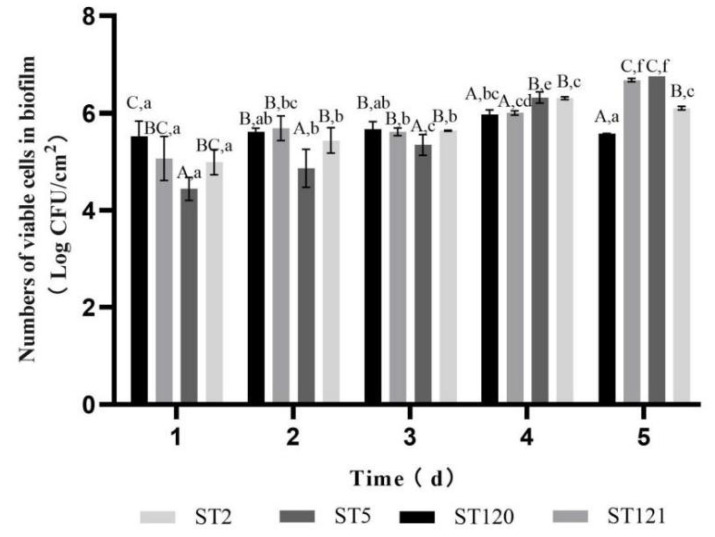
Biofilm formation ability of four ST isolates. The lowercases represent the difference among the same ST at different times. The capitals represent the difference among four ST at the same time.

It can be obtained that the biofilm formation ability of the four STs isolates was different (Figure 3). In the first three days, the enumeration of biofilm cells of ST5 was lower than that of the other three ST isolates, and *p*-values were 0.002, 0.000, and 0.000 for ST5 relative to ST2, ST120, and ST121, respectively. However, after the third day, the number of biofilm cells of the ST5 isolate increased, which was higher than those of the ST121 and ST120 isolates. On the fifth day, the number of biofilm cells formed by ST5 and ST121 was significantly higher than those of the ST120 and ST2 isolates (*p* < 0.05), the *p*-values were 0.012 and 0.000 for ST5 relative to ST2 and ST120, while the *p*-values were 0.018 and 0.001 for ST121 relative to ST2 and ST120, respectively.

### 3.4. Disinfection Efficiencies of Chlorine-Containing Disinfectant against Different ST Biofilm Cells and Disinfectant-Resistant Genes

The four different ST isolates had different disinfectant-resistant genes. Three disinfectant-resistant genes had been identified. ST5 and ST121 had the three genes *clpL*, *mdrL*, and *lde*; however, ST120 and ST2 had *mdrL* and *lde* but not *clpL*. All four STs did not have *qacH* (Table 1).

In this study, three concentrations of chlorine-containing disinfectant were applied: 500 mg/L, 250 mg/L, and 125 mg/L. The biofilm cells growing for 96 h were used for disinfection efficiencies. The initial concentration of biofilm cells of four ST *L. monocytogenes* was between 6.08 and 6.36 log. The acting time of chlorine containing was 30 s and 60 s, respectively. The disinfection efficiency of different concentrations of chlorine-containing disinfectant to *L. monocytogenes* biofilm cells was different (Table 2). When the three concentrations (500 mg/L, 250 mg/L, and 125 mg/L) of chlorine-containing disinfectant were used on ST5, the reduction values of biofilm cells were 6.32 log, 4.82 log, and 3.34 log respectively, and the difference in reduction values was significant (*p* < 0.05). When the three concentrations (500 mg/L, 250 mg/L, and 125 mg/L) of chlorine-containing disinfectant were used on ST121 and ST5, the reduction values of biofilm cells reduced; however, there were no significant differences. When the three concentrations (500 mg/L, 250 mg/L, and 125 mg/L) of chlorine-containing disinfectant were used on ST120, the disinfection efficiency was 100% with reduction values of 6.08 log.

Alternatively, the disinfection efficiency of the same concentration of chlorine-containing disinfectant was different (Table 2). When 500 mg/L chlorine-containing disinfectant was used, the disinfection efficiency of chlorine-containing disinfectant to the four STs biofilm cells was 100%. However, when 250 mg/L chlorine-containing disinfectant was used, the reduction values of ST120 and ST2 biofilm cells were 6.08 log and 6.36 log, respectively, with 100% disinfection efficiency. In contrast, the reduction values of ST5 and ST121 were 4.82 log and 5.84 log. Furthermore, when 125 mg/L chlorine-containing disinfectants were used, only the disinfection efficiency of chlorine-containing disinfectant on ST120 was 100%. The reduction values of ST5 and ST121 biofilm cells were 3.34 log and 4.94 log. Thus, the difference between disinfection efficiency of ST5, ST121 and ST120, ST2 was significant (*p* < 0.05).

## 4. Discussion

The complete genome sequences of several *L. monocytogenes* strains have showed a high degree of similarity in gene content and organization [16,17,18], which suggested that the genomic compositions of different *Listeria* strains are highly stable, and the number of genes that play essential roles in the ability to adapt to environments may be relatively small [16,17,28]. Mechanisms that promote the persistence of *L. monocytogenes* isolates in food processing environments include biofilm formation [29,30], resistance to disinfectants [31,32], and stress tolerance mechanisms [33]. Therefore, in this study, these genes, plasmids and prophages have been compared in the 22 STs *L. monocytogenes* isolates to analyze their capacity in adapting to environments. To date, six genes (*mdrL*, *lde*, cassette bcr*ABC*, *qacH*, *emrE*, and *emrC*), located on mobile genetic elements, associated with the efflux system, have been identified in *L. monocytogenes* in response to disinfectant [34]. The two major efflux pump genes, *mdrL* and *lde*, are present in all four ST isolates in this study, which is consistent with previous study; these two efflux pump genes are present in all *L. monocytogenes* [35]. The other four efflux pump genes are present in four STs in this study. These results suggested that there might be other genes affecting the response to disinfectant action.

SSI-1 was associated with tolerance to acidic, bile, gastric and salt stresses and has been reported to be prevalent in ST9, ST204 and ST321 [34]. In this study, SSI-1 has been identified in ST5, ST121 and ST120. This result suggested that ST5, ST121 and ST120 might be more tolerant of food processing environments than ST2.

The biofilm-forming ability of ST120 was higher than that of the other three STs (ST5, ST121 and ST2) when grown for one day (24 h) (Figure 1). The analysis of biofilm formation-associated genes indicated that ST120 owned all six biofilm-associated genes. However, the other three STs had fewer biofilm formation genes. Furthermore, the three STs isolates do not have *actA*. The *actA* gene is present in ST120. This *actA* gene is known to be responsible for the actin polymerization, which is essential for the movement of *L. monocytogenes* within the host cell and for the first step of biofilm formation [36,37]. It has been reported that the ST2 isolates have a lower ability of biofilm formation because ST2 isolates had fewer overall biofilm formation-associated genes, especially missing a vital biofilm-forming *actA* gene [38]. These results indicated that *actA* might play a vital role in biofilm formation by regulating the movement of *L. monocytogenes* in host cells in *L. monocytogenes*. Alternatively, the InlA protein has been reported to be critical for adhesion and host cell invasion, which has been implicated in biofilm formation. Research by Olier at al. proposed that truncation of the InlA-encoding gene *inlA* significantly enhances biofilm [39]. However, this conclusion is not supported by the observation provided by Wang [30]. Additionally, in this study, ST2 with truncated *inlA* showed lower biofilm formation ability than that of ST120 when grown over one day; then, it exhibited higher biofilm formation ability than that of the ST120 when grown for four days. These results suggested that *inlA* and truncated *inlA* played complex roles in biofilm formation in *L. monocytogenes* in addition to other factors (e.g., acid, nutrients etc.). These results support the idea that biofilm formation ability is primarily a random process. Additionally, most *L. monocytogenes* isolates can show stronger biofilm formation ability if present in an appropriate niche at the proper time.

Note that the biofilm-forming ability of ST5-IIb and ST121-IIa increased after being grown for four days. However, the biofilm formation ability of ST2-IVb and ST120-IIa reduced after growing for four days. The poor biofilm-forming ability of IVb *L. monocytogenes* isolates, where nutrients may be limited, has been reported [38]. In this study, when grown for four days, the nutrients necessary for *L. monocytogenes* would be limited, and hazards for microorganisms would be increasing. As a result, the unfavorable environment would affect the biofilm-forming ability of *L. monocytogenes*. Furthermore, only ST5 and ST121 own the *clpL* gene, which encodes ClpL. The ClpL protein has been found in some Listeria plasmids before [40]. Under various conditions in *Streptococcus* spp. and *Lactobacillus* spp., highly similar (approximately 68% amino acid identity) ClpL proteins are involved in stress response and act as a chaperone for the stress response regulator CtsR [41,42,43]. Therefore, ST5 and ST121 would be more tolerant to disadvantageous environments. These results suggest that the ability of tolerance to environments also plays an important role in biofilm formation, affecting the persistence of *L. monocytogenes* in environments.

It is worthwhile to note the potential persistence of ST121 in food processing environments. ST121 had a similar biofilm formation ability with ST5 and resistance to chlorine-containing disinfectant in this study. Additionally, SSI-1 was predominantly found in food-associated strains of ST121, which were consistent with the results in this study. SSI-2 is involved in the alkaline and oxidative stress response [44]. The ST121 isolates are frequently found to be abundant and persist in food processing environments. They have specific genetic determinants that support persistence and confer adaptation to a taxonomically discriminative niche [45]. Therefore, stronger surveillance should be conducted in food processing plants especially in RTE food processing environments.

In food production environments, bacteria are regularly exposed to cleaning and disinfectants. The efficient cleaning and sanitization processes are essential to prevent food contamination by *L. monocytogenes* during processing. However, the disinfection process is not always performed adequately. *L. monocytogenes* isolates are often exposed to sub-inhibitory concentrations of disinfectants due to dilution in the environment and biodegradation, resulting in a gradient of disinfectants concentration. Furthermore, *L. monocytogenes* isolates residing in food processing environments may adapt to disinfectant after repeated exposure. As a result, the low-level resistance to disinfectants may contribute to its environment adaptation and persistence [46]. Presently, few studies have investigated how different concentrations of disinfectant affect different STs *L. monocytogenes* isolates. To our knowledge, this is the first study showing the effect of different concentrations of chlorine-containing disinfectant acting on different STs isolates and differences of relative genes among the four ST *L. monocytogenes* isolates in this study. The three concentrations of chlorine-containing disinfectant (500 mg/L, 250 mg/L, and 125 mg/L) were applied in this study. The 500 mg/L concentration was proposed to be lethal concentration according to the manufacturers. The results showed that only ST2 biofilm cells had been entirely cleaned by all three concentrations of chlorine-containing disinfectants. Conversely, ST5 and ST121 biofilm cells survive significantly after acting with 125 mg/L and 250 mg/L chlorine-containing disinfectant. ST5, ST121 and ST2 have different disinfectant-resistant genes. ST5 and ST121 had all three genes (*clpL*, *mdrL* and *lde*), whereas ST2 had only *mdrL* and *lde*. The *clpL* gene has been involved in stress response under various conditions [41,42,43,47]. These results suggested that the gene *clpL* might play an essential role in resistance to chlorine-containing disinfectants. Therefore, from a practical perspective, the study emphasizes that disinfectants should be used at the lethal concentrations recommended by the manufacturers. Further studies are required to elucidate the lethal concentration of chlorine-containing disinfectants in food processing environments.

## 5. Conclusions

The ST5 *L. monocytogenes* isolates have likely persisted in an RTE meat processing plant for a long time in Shanghai, China. Biofilm formation has contributed to this bacterial persistence. Our results suggested that the biofilm formation ability of *L. monocytogenes* isolates might be different at different stages. Furthermore, the *actA* gene might play an important role at the beginning of biofilm formation. However, the biofilm formation ability of ST5 and ST121 isolates, which do not have *actA*, increased significantly after grown for four days. Simultaneously, ST5 and ST121 biofilm cells have stronger resistance ability to sub-lethal chlorine-containing disinfectants. The result suggested that disinfectants should be used at the lethal concentrations recommended by the manufacturers.

This study helped understand the mechanisms of *L. monocytogenes* persistence in RTE food processing environments and provided a theoretical reference for taking measures to prevent and control the transmission of *L. monocytogenes* in food processing environments.

## Figures and Tables

**Table 1:** Genetic features of four STs *L. monocytogenes* from the two RTE meat food processing plants from 2019 to 2020.

ST	Serogroup	Disinfectant Resistant Genes	Stress Survival Ilset	*inlA*	*qacH*, *ermE*, *ermC*	Plasmids	Intact Prophages	Biofilm Formation-Related Genes					
								*actA*	*prfA*	*lmo0673*	*recO*	*lmo2504*	*luxS*
2	IVb	*mdrL*, *lde*	*-*	truncated *inlA*	*-*	pLM33 pLM5578	B025_NC_ 009812	*−*	+	+	+	*−*	*−*
5	IIb	*clpL*, *mdrL*, *lde*	SSI-1	*inlA*	*-*	pLM33	B025_NC_ 009812	*−*	+	+	+	+	+
120	IIa	*mdrL*, *lde*	SSI-1	*inlA*	*-*	*-*	*-*	+	+	+	+	+	+
121	IIa	*clpL*, *mdrL*, *lde*	SSI-1, SSI-2	*inlA*	*-*	pLM5578	*-*	*−*	*−*	+	+	+	+

Note: “−” means the genetic feature is not present, ”+” means the genetic feature is present.

**Table 2:** Disinfection efficiency of chloride-containing disinfectant to four ST isolates.

Strains	Disinfectant Concentration (mg/L)	Log Reduction Values (LogCFU/cm^2^) and Disinfection Efficiency (%)				
		Disinfectant Treatment Time (s)				
		0	30		60	
ST120	125	6.08 ± 0.06 ^A^	3.17 ± 0.04 ^Ba^	52.04%	6.08 ± 0.06 ^Ba^	100.00%
	250		4.01 ± 0.04 ^Bb^	65.94%	6.08 ± 0.06 ^Aa^	100.00%
	500		6.08 ± 0.06 ^Ac^	100.00%	6.08 ± 0.06 ^Aa^	100.00%
ST121	125	6.31 ± 0.23 ^A^	3.13 ± 0.14 ^Ba^	49.69%	4.94 ± 0.97 ^ABa^	78.27%
	250		3.36 ± 0.04 ^Ab^	57.40%	5.84 ± 0.71 ^Aa^	92.63%
	500		6.31 ± 0.23 ^Ac^	100.00%	6.31 ± 0.23 ^Aa^	100.00%
ST5	125	6.32 ± 0.03 ^A^	2.84 ± 0.09 ^Aa^	44.93%	3.34 ± 0.62 ^Aa^	52.85%
	250		3.83 ± 0.12 ^ABb^	60.63%	4.82 ± 1.28 ^Abc^	76.17%
	500		6.32 ± 0.03 ^Ac^	100.00%	6.32 ± 0.03 ^Ac^	100.00%
ST2	125	6.36 ± 0.03 ^A^	3.64 ± 0.12 ^Ca^	57.27%	5.54 ± 1.40 ^ABa^	87.04%
	250		6.36 ± 0.03 ^Cb^	100.00%	6.36 ± 0.03 ^Aa^	100.00%
	500		6.36 ± 0.03 ^Ab^	100.00%	6.36 ± 0.03 ^Aa^	100.00%

Note: the capitals represent differences of disinfection efficiency among different STs acted by the same concentrations of chloride-containing disinfectant. The lowercases represent differences of disinfection efficiency of the same ST acted by different concentrations of chloride-containing disinfectant.

## Data Availability

Data is contained within the article, more details are available from the corresponding author.

## References

[1] Allerberger F., Wagner M. (2010). Listeriosis: A resurgent foodborne infection. Clin. Microbiol. Infect..

[2] Lianou A., Sofos J.N. (2007). A review of the incidence and transmission of *Listeria monocytogenes* in ready-to-eat products in retail and food service environments. J. Food Prot..

[3] Zhang H.Z., Que F.X., Xu B., Sun L., Zhu Y., Chen W., Ye Y., Dong Q., Liu H., Zhang X. (2021). Identification of *Listeria monocytogenes* contamination in a ready-to-eat meat processing plant in China. Front. Microbiol..

[4] Orsi R.H., Borowsky M.L., Lauer P., Young S.K., Nusbaum C., Galagan J.E., Birren B.W., Ivy R.A., Sun Q., Graves L.M. (2008). Short-term genome evolution of *Listeria monocytogenes* in a non-controlled environment. BMC Genom..

[5] Tompkin R.B. (2002). Control of *Listeria monocytogenes* in the food-processing environment. J. Food Prot..

[6] Zhang H.Z., Wang J., Chang Z.Y., Liu X., Chen W., Yu Y., Wang X., Dong Q., Ye Y., Zhang X. (2021). *Listeria monocytogenes* contamination characterization in two ready-to-eat meat plants from 2019 to 2020 in Shanghai. Front. Microbiol..

[7] Gandhi M., Chikindas M.L. (2007). Listeria: A foodborne pathogen that knows how to survive. Int. J. Food Microbiol..

[8] Kim J.W., Kathariou S. (2009). Temperature-dependent phage resistance of *Listeria monocytogenes* epidemic clone II. Appl. Environ. Microbiol..

[9] Fox E.M., Allnutt T., Bradbury M.I., Fanning S., Chandry P.S. (2016). Comparative genomics of the *Listeria monocytogenes* ST204 subgrou. Front. Microbiol..

[10] Knudsen G.M., Nielsen J.B., Marvig R.L., Ng Y., Worning P., Westh H., Gram L. (2017). Genome-wide-analyses of *Listeria monocytogenes* from food-processing plants reveals clonal diversity and dates the emergence of persisting sequence types. Environ. Microbiol. Rep..

[11] Maury M.M., Tsai Y., Charlier C., Touchon M., Chenal-Francisque V., Leclercq A., Criscuolo A., Gaultier C., Roussel S., Brisabois A. (2016). Uncovering *Listeria monocytogenes* hypervirulence by harnessing its biodiversity. Nat. Genet..

[12] Moura A., Criscuolo A., Pouseele H., Maury M.M., Leclercq A., Tarr C., Björkman J.T., Dallman T., Reimer A., Enouf V. (2016). Whole genome-based population biology and epidemiological surveillance of *Listeria monocytogenes*. Nat. Microbiol..

[13] Annabel L.N., Monika D., Martin W., Schmitz-Esser S. (2019). Plasmids contribute to food processing environment-associated stress survival in three *Listeria monocytogenes* ST121, ST8, and ST5 strains. Int. J. Food Microbiol..

[14] Muhterem-Uyar M., Ciolacu L., Wagner K.H., Wagner M., Schmitz-Esser S., Stessl B. (2018). New Aspects on *Listeria monocytogenes* ST5-ECVI Predominance in a Heavily Contaminated Cheese Processing Environment. Front. Microbiol..

[15] Wang G., Qian W., Zhang X., Wang H., Ye K., Bai Y., Zhou G. (2015). Prevalence, genetic diversity and antimicrobial resistance of *Listeria monocytogenes* isolated from ready-to-eat meat products in Nanjing, China. Food Control..

[16] Jacquet C., Doumith M., Glaser P., Martin P., Buchrieser C. (2004). Differentiation of the major *Listeria monocytogenes* serovars by multiplex PCR. J. Clin. Microbiol..

[17] Doumith M., Cazalet C., Simoes N., Frangeul L., Jacquet C., Kunst F., Martin P., Cossart P., Glaser P., Buchrieser C. (2004). New aspects regarding evolution and virulence of *Listeria monocytogenes* revealed by comparative genomics and DNA arrays. Infect. Immun..

[18] Nelson K.E., Fouts D.E., Mongodin E.F., Ravel J., DeBoy R.T., Kolonay J.F., Rasko D.A., Angiuoli S.V., Gill S.R., Paulsen I.T. (2004). Whole genome comparisons of serotype 4b and 1/2a strains of the food-borne pathogen *Listeria monocytogenes* reveal new insights into the core genome components of this species. Nucleic Acids Res..

[19] Gray M.J., Zadoks R.N., Fortes E.D., Dogan B., Cai S., Chen Y., Scott V.N., Gombas D.E., Boor K.J., Wiedmann M. (2004). *Listeria monocytogenes* isolates from foods and humans form distinct but overlapping populations. Appl. Environ. Microbiol..

[20] Barbour A.H., Rampling A., Hormaeche C.E. (2001). Variation in the infectivity of *Listeria monocytogenes* isolates following intragastric inoculation of mice. Infect. Immun..

[21] Ryan S., Begley M., Hill C., Gahan C.G. (2010). A five-gene stress survival islet (SSI-1) that contributes to the growth of *Listeria monocytogenes* in suboptimal conditions. J. Appl. Microbiol..

[22] Sela S., Frank S., Belausov E., Pinto R. (2006). A mutation in the luxS gene influences *Listeria monocytogenes* biofilm formation. Appl. Environ. Microbiol..

[23] Burall L.S., Grim C.J., Mammel M.K., Datta A.R. (2015). Whole genome sequence analysis using JSpecies tool establishes clonal relationships between *Listeria monocytogenes* strains from epidemiologically unrelated listeriosis outbreaks. PLoS ONE.

[24] Ragon M., Wirth T., Hollandt F., Lavenir R., Lecuit M., Le Monnier A., Brisse S. (2008). A new perspective on *Listeria monocytogenes* evolution. PLoS Pathog..

[25] Arndt D., Grant J.R., Marcu A., Sajed T., Pon A., Liang Y., Wishart D.S. (2016). PHASTER: A better, faster version of the PHAST phage search tool. Nucleic Acids Res..

[26] Schmita-Esser S., Muller A., Stessl B., Wagner M. (2015). Genomes of sequence type 121 *Listeria monocytogenes* strains harbor highly conserved plasmids and prophages. Front. Microbiol..

[27] Kostoglou D., Protopappas I., Giaouris E. (2020). Common plant-derived terpenoids present increased anti-biofilm potential against Staphylococcus bacteria composed to a quaternary ammonium biocide. Foods.

[28] Buchrieser C., Rusniok C., Listeria C.T., Kunst F., Cossart P., Glaser P. (2003). Comparison of the genome sequences of *Listeria monocytogenes* and *Listeria innocua*: Clues for evolution and pathogenicity. FEMS Immunol. Med. Microbiol..

[29] Nakamura H., Takakura K., Sone Y., Itano Y., Nishikawa Y. (2013). Biofilm formation and resistance to benzalkonium chloride in *Listeria monocytogenes* isolated from a fish processing plant. J. Food Prot..

[30] Wang J., Ray A.J., Hammons S.R., Oliver H.F. (2015). Persistent and transient *Listeria monocytogenes* strains from retail deli environments vary in their ability to adhere and form biofilms and rarely have inlA premature stop codons. Foodborne Pathog. Dis..

[31] Müller A., Rychli K., Muhterem-Uyar M., Zaiser A., Stessl B., Guinane C.M., Cotter P.D., Wagner M., Schmitz-Esser S. (2013). Tn6188-a novel transposon in *Listeria monocytogenes* responsible for tolerance to benzalkonium chloride. PLoS ONE.

[32] Fox F.M., Leonard N., Jordan K. (2011). Physiological and transcriptional characterization of persistent and nonpersistent *Listeria monocytogenes* isolates. Appl. Environ. Microbiol..

[33] Cabrita P., Trigo M.J., Ferreira R.B., Brito L. (2015). Differences in the expression of cold stress-related genes in the swarming motility among persistent and sporadic strains of *Listeria monocytogenes*. Foodborne Pathog. Dis..

[34] Wiktorczyk-Kapischke N., Skowron K., Grudlewska-Buda K., Wałecka-Zacharska E., Korkus J., Gospodarek-Komkowska E. (2021). Adaptive Response of *Listeria monocytogenes* adaptive response to the stress factors in the food processing environment. Front. Microbiol..

[35] Haubert L., Zehetmeyr M.L., da Silva W.P. (2019). Resistance to benzalkonium chloride and cadmium chloride in *Listeria monocytogenes* isolates from food and food processing environments in southern Brazil. Can. J. Microbiol..

[36] Mafuna T., Matle I., Magwedere K., Pierneef R.E., Reva O.N. (2021). Whole genome-based characterization of *Listeria monocytogenes* isolates recovered from the food chain in South Africa. Front. Microbiol..

[37] Pasquali F., Palma F., Guillier L., Lucchi A., Lucchi A., De Cesare A., Manfreda G. (2018). *Listeria monocytogenes* sequence types 121 and 14 repeatedly isolated within one year of sampling in a rabbit meat processing plant: Persistence and ecophysiology. Front. Microbiol..

[38] James P.F., Gregory R.S., Joseph F.F. (2006). Formation of biofilm at different nutrient levels by various genotypes of *Listeria monocytogenes*. J. Food Protect..

[39] Olier M., Pierre F., Rousseaux S., Lamaitre J.P., Rousset A., Piveteau P., Guzzo J. (2003). Expression of truncated internalin A is invovled in impaired internalization of some *Listeria monocytogenes* isolates carried asymptomatically by humans. Infect. Immun..

[40] Kuenne C., Billion A., Mraheil M.A., Strittmatter A., Daniel R., Goesmann A., Strittmatter A., Daniel R., Goesmann A., Barbuddhe S. (2013). Reassessment of the *Listeria monocytogenes* pan-genome reveals dynamic integration hotspots and mobile genetic elements as major components of the accessory genome. BMC Genom..

[41] Suokko A., Savijoki K., Malinen E., Palva A., Varmanen P. (2005). Characterization of a mobile *clpL* gene from *Lactobacillus rhamnosus*. Appl. Environ. Microbiol..

[42] Kajfasz J.K., Martínez A.R., Rivera-Ramos I., Abranches J., Koo H., Quivey R.G., Lemos J.A. (2009). Role of Clp proteins in expression of virulence properties of Streptococcus mutans. J. Bacteriol..

[43] Tran T.D., Kwon H.Y., Kim E.H., Kim K.W., Briles D.E., Pyo S., Rhee D.K. (2011). Decrease in penicillin susceptibility due to heat shock protein ClpL in Streptococcus pneumoniae. Antimicrob Agents Chemother..

[44] Harter E., Wagner E.M., Zaiser A., Halecker S., Wagner M., Rychli K. (2017). Stress survival islet 2, predominantly present in *Listeria monocytogenes* strains of sequence type 121, is involved in the alkaline and oxidative stress responses. Appl. Environ. Microbiol..

[45] Hein I., Klinger S., Dooms M., Flekna G., Stessl B., Leclercq A., Hill C., Allerberger F., Wagner M.J.A., Microbiology E. (2011). Stress survival islet (SSI-1) survey in *Listeria monocytogenes* reveals an insert common to *Listeria innocua* in sequence type 121 *L. monocytogenes* strains. Appl. Environ. Microbiol..

[46] Martínez-Suárez J.V., Ortiza S., López-Alonso V. (2016). Potential impact of the resistance to quanternary ammonium disinfectants on the persistence of Listeria monocytogenes in food processing environments. Front. Microbiol..

[47] Tao L., Biswas I. (2013). ClpL is required for folding of CtsR in Streptococcus mutans. J. Bacteriol..

